# Management of Flabby Edentulous Ridge Using the Window Impression Technique

**DOI:** 10.7759/cureus.41340

**Published:** 2023-07-03

**Authors:** Shreya Colvenkar, Vishnu Priya Cherukuri, Sunitha J D., Ravindra S V., Jayasri Vanapalli

**Affiliations:** 1 Department of Prosthodontics, Manthena Narayana Raju Dental College and Hospital, Sangareddy, IND; 2 Department of Orthodontics, Manthena Narayana Raju Dental College and Hospital, Sangareddy, IND; 3 Department of Oral Pathology and Microbiology, Manthena Narayana Raju Dental College and Hospital, Sangareddy, IND; 4 Department of Oral Medicine and Radiology, Manthena Narayana Raju Dental College and Hospital, Sangareddy, IND

**Keywords:** impression plaster, impression, window, edentulous, flabby

## Abstract

Flabby ridge, also known as a mobile or displaceable ridge, is a common clinical challenge encountered in dental practice. The flabby ridge lacks firmness and stability, causing dentures to become loose and unstable. This can lead to discomfort, difficulty in speaking and eating, and a decreased quality of life for denture wearers. The flabby ridge often lacks the necessary contour and resilience to create an effective seal and maintain suction between the denture base and the underlying tissues. This compromises the retention of dentures, making them prone to dislodgement during normal oral functions. In this report, a modified window technique is presented for the impression of the anterior maxillary flabby ridge. The window ensures that no or little pressure is exerted on the flabby tissue. Therefore, this technique can be easily carried out by general dental practitioners, enabling them to manage flabby ridge complete denture cases within a primary dental care setting.

## Introduction

Flabby ridge, also known as a mobile or displaceable ridge, is a common clinical challenge encountered in dental practice. It refers to the excessive soft tissue found in an edentulous area, which often leads to poor denture retention, support, and stability. According to several studies [1], approximately 5% of edentate mandibles and 24% of edentate maxillae exhibit flabby ridges.

Various surgical interventions are employed for managing flabby ridges, including scalpel surgery to remove excess soft tissue or the use of a sclerosing agent before complete denture fabrication [2]. Moreover, surgical ridge augmentation techniques are also suggested as potential methods for addressing flabby ridges. However, surgical excision of flabby tissue results in increased denture material bulk and the loss of stress-absorbing soft tissues, which can potentially cause trauma to the underlying tissues [3]. 

As a result, conventional prosthodontic approaches, including special impression techniques and the balancing of occlusal loads, are more commonly employed in the management of flabby ridges. This article presents a case report for the prosthodontic rehabilitation of a patient with a flabby ridge using the window impression technique. 

## Case presentation

**Figure 1:**
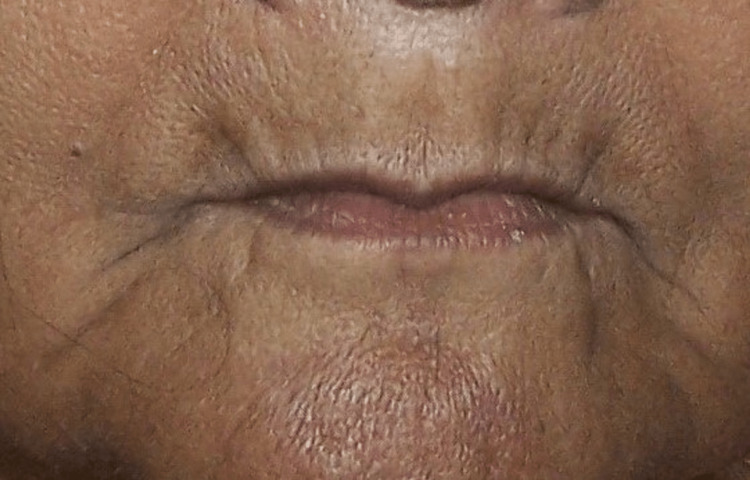
Pre-treatment photograph.

A 70-year-old elderly, the completely edentulous patient reported to the Department of Prosthodontics, M. N. Raju Dental College and Hospital, Sangareddy, India, for the fabrication of new dentures. The maxillary impression tray was modified by making a window in the flabby area (Figure 1).

**Figure 2:**
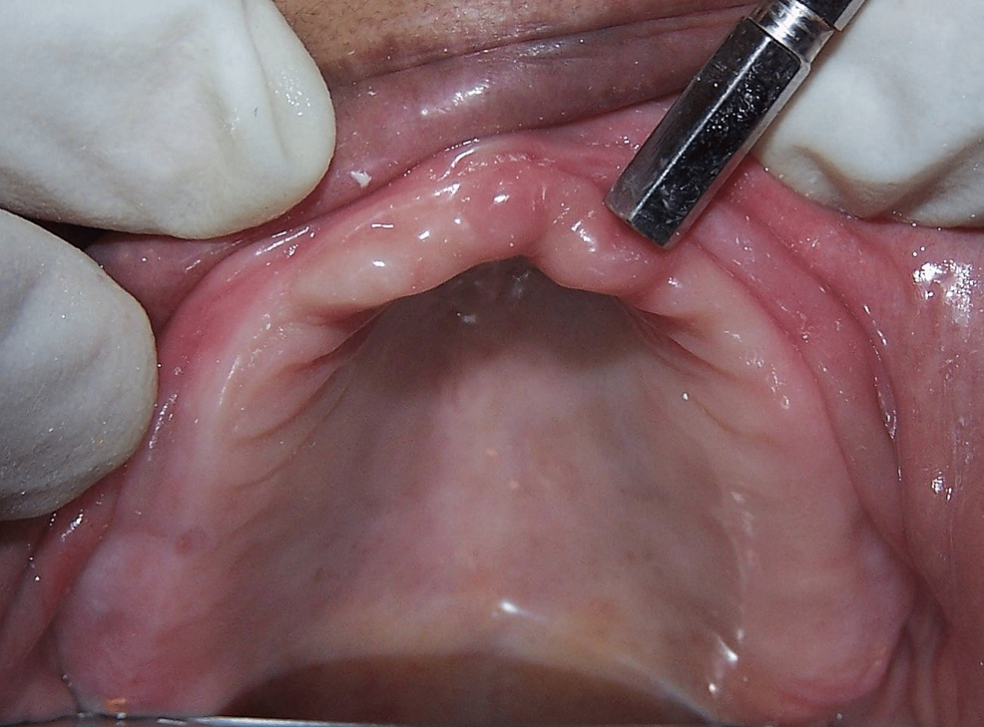
Flabby anterior maxillary edentulous ridge.

Intraoral examination revealed the presence of excessive flabby tissue in the anterior part of the maxillary residual alveolar ridge (Figure 2).

**Figure 3:**
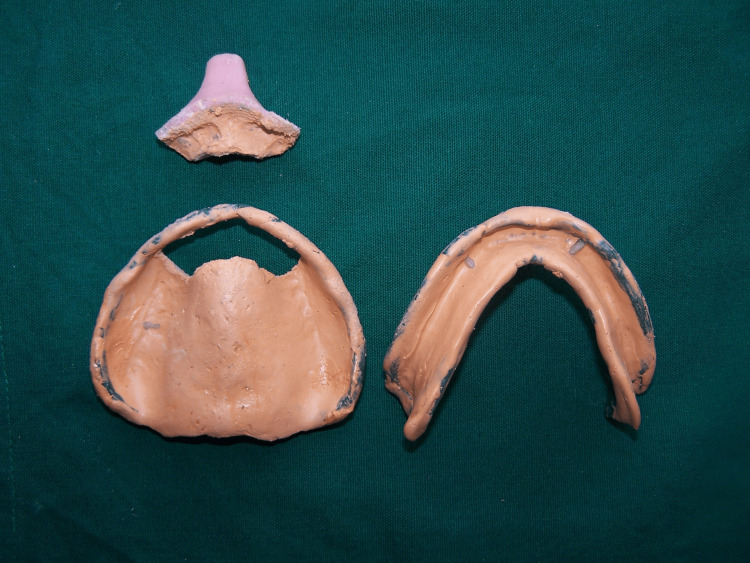
Window impression technique.

To minimize the displacement of the mobile tissue, primary impressions were made with irreversible hydrocolloid (Chromatex, DPI, India) in a perforated totally edentulous stock tray. A custom tray was fabricated using auto-polymerizing acrylic resin (DPI cold cure; Dental Products of India, Mumbai, India) using the selective pressure impression theory, and the tray's borders were reduced to be 2 mm short of the sulcus. Border molding was performed using a low-fusing impression compound (DPI Pinnacle, Tracing Sticks Dental Products of India). The spacer was then removed, and the definitive impression was made using zinc oxide eugenol impression paste. Following the border molding and secondary impression, a window was created in the custom tray in the flabby tissue area using a round and fissured bur (Figure 3).

**Figure 4:**
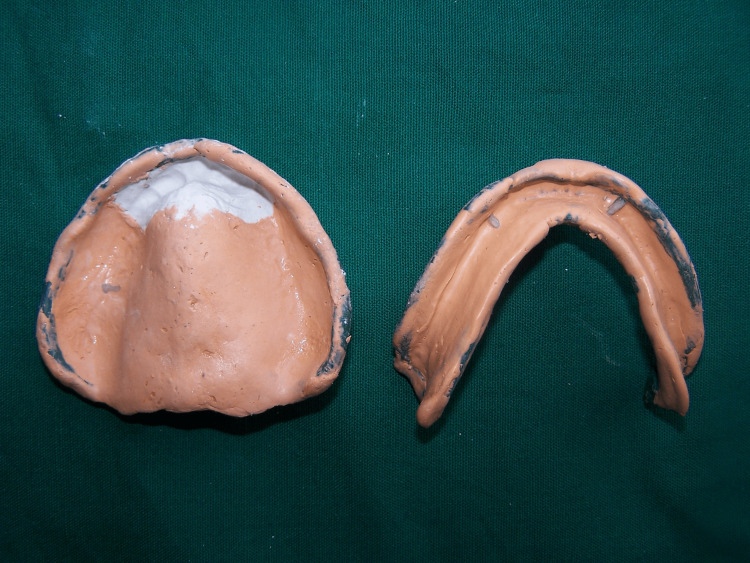
Final impression with impression plaster.

The tray was checked in the mouth to verify if the flabby area was visible through the window. This was followed by a coating of impression plaster (Kalabhai, India) in the window region using a gentle paintbrush. A custom tray was again inserted in the patient’s mouth (Figure 4).

**Figure 5:**
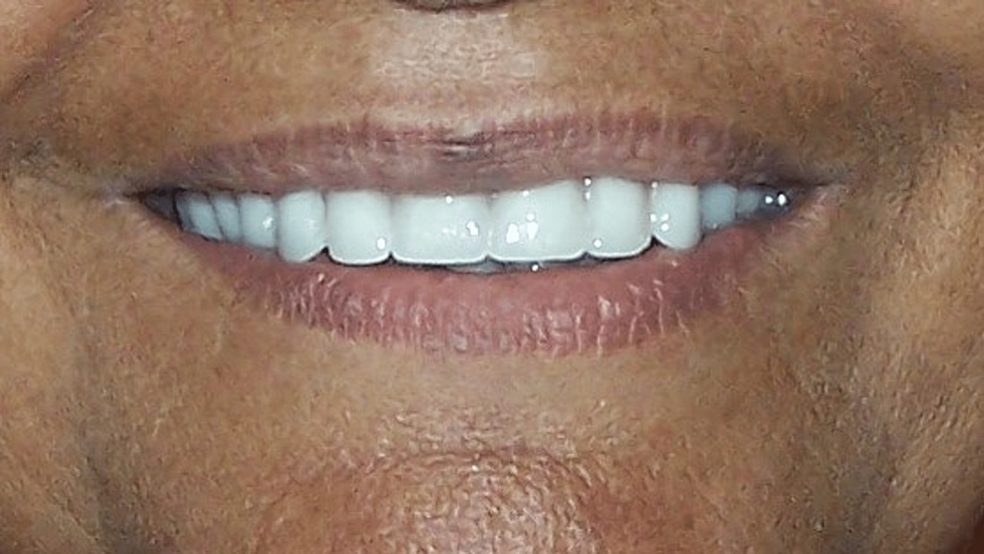
Post-treatment photograph.

Once the material had been set, the custom impression tray was removed from the mouth. After using the soap solution as a separator over the impression, the master cast was poured. Rest all steps of complete denture fabrication were completed according to conventional procedure. Complete dentures were successfully delivered to the patient after post-insertion instructions (Figure 5).

## Discussion

The compression of flabby ridges during conventional impression techniques can lead to instability, loss of denture retention, and dislodgement due to the elastic recoil of the fibrous soft tissue during function [2]. While individualized treatment strategies are often required for cases involving flabby ridges, a comprehensive change in the overall treatment plan is crucial. Upon reviewing the existing literature, it becomes evident that the majority of modifications in the conservative management of flabby ridges revolve around impression techniques and materials. In addition to the ongoing debate between mucostatic and selective impression techniques, the literature also highlights the use of modified or alternative techniques and the application of different materials [4]. Various methods such as spacers or perforations [5], sectional trays, or split trays [6,7] have also been reported. However, there is currently no evidence to support the superiority of one particular technique over others in providing a stable and retentive denture on flabby ridges [4].

In this report, a modified window technique is presented for the impression of the anterior maxillary flabby ridge. The technique stated in this case is virtually identical to the technique described by Watson [8]. A mucocompressive/selective pressure impression technique is utilized on normal tissues, while the impression plaster over the window records the tissue in a static state. Liddelow first proposed the use of zinc oxide, eugenol, and impression plaster in 1964 [9]. The choice of window is possibly the greatest way to ensure that no or little pressure is exerted to the flabby tissue. Recent research on several tray designs has discovered that the tray with a window creates the fewest tissue variations [10]. The use of impression plaster during the impression process can lead to tissue compression or displacement. However, incorporating a window in the tray design prevents the plaster from being confined within the tray. This allows the flabby tissue to fully rebound while the plaster is setting.

Furthermore, the clear tray provides visibility for clinicians, allowing them to observe the adaptation of the impression material to the flabby tissue. Recording the flabby area in a rest state, without displacement or compression, is crucial. The window has the additional advantage of allowing the flabby tissue to recoil back to its resting position, even if some pressure has been applied. Therefore, it is essential to use a window and impression materials such as impression plaster and light body to accurately record the flabby ridge in a rest state.

## Conclusions

Techniques for managing flabby ridges involve a combination of approaches aimed at improving denture stability, retention, support, and patient comfort. The selection of the appropriate impression technique depends on factors such as clinical presentation, patient comfort, operator experience, and the availability of resources. A thorough assessment of the individual case is necessary to determine the most suitable impression technique for managing flabby ridges. This report presents a window technique that showcases a reliable method for the controlled application of impression plaster material, ensuring a final impression of the flabby ridge without any displacement. The window ensures that no or little pressure is exerted on the flabby tissue. This technique requires additional time to create an accurate window for impression but does not necessitate additional clinical visits.
